# Supplementary material for: Differential Immunomodulatory Effect of Graphene Oxide and Vanillin-Functionalized Graphene Oxide Nanoparticles in Human Acute Monocytic Leukemia Cell Line (THP-1)
Source: Int J Mol Sci. 2019 Jan 10;20(2):247. doi: 10.3390/ijms20020247 (PMC6359521; doi:10.3390/ijms20020247)
Supplement: Supplementary file 1 [file ijms-20-00247-s001.pdf]

Table S1

Gene	List of primers
**APEX1**	F:ATTGGCTGGAGGGCAGATCT
	R:CCACTGGGTGAGGTTTTCTGA
**OGG1**	F:TCCTCCCTAGGTTTCCTCTC
	R:TGAGACTAGTGACAGTGTTGG
**P53**	F:AGAGACCGTACAGAAGA
	R:CTGTAGCATGGGATCCTTT
**P21**	F:GTTGCTGTCCGGACTACCG
	R:AAAAACAATGCCACCACTCC
**Caspase-3**	F:AGGGGTCATTTATGGGACA
	R:TACACGGGATCTGTTTCTTTG
**Caspase-9**	F:GTCACGGCTTTGATGGAGAT
	R:CAGGCCTGGATGAAGAAGAG
**Bax**	F:CGAGCTGATCAGAACCATCA
	R:GAAAAATGCCTTTCCCCTTC
**POLB**	F:GTTTCAGAAGAGGTGCAGAG
	R:AGTGAAATAGAGAACACCACAG
**Bcl-2**	F:TAAGCTGTCACAGAGGGGCT
	R:TGAAGAGTTCCTCCACCACC
**CREB1**	F:CAGTTCAGTCTTCCTGTAAGGACT
	R:CGTTTGTCATGGTTAGTGTC
**UNG**	F:CTCTGCTTTAGTGTTCAAAGG
	R:GAGTTCTGATTTAGCCAGGA
	R:CCTTTGTACCGTTGCATCCT
**GAPDH**	F:AGGTCGGTGTGAACGGATTTG
	R:TGTAGACCATGTAGTTGAGGTCA
